# Effects of *TRIB3* gene overexpression and interference on lipid metabolism in mammary epithelial cells of yaks

**DOI:** 10.3389/fvets.2026.1774228

**Published:** 2026-05-05

**Authors:** Jiale Zheng, Fan Lei, Shiang Liu, Yaqin Zhao, Ziyu Zhang, Baoxia Dong, Huina Feng, Haixia Jing

**Affiliations:** 1 College of Agriculture and Animal Husbandry, Qinghai University, Xining, China; 2 Jinshan Animal Husbandry and Veterinary Station of Lantian County, Xian, Shaanxi, China; 3 Hualong County Bureau of Agriculture, Rural and Science and Technology, Haidong, Qinghai, China

**Keywords:** interference, milk fat metabolism, overexpression, TRIB3, YMECs

## Abstract

*TRIB3* often acts as a negative regulator of lipid metabolism in the body, however, little is known about the effect of *TRIB3* on milk lipid metabolism in YMECs. Therefore, we investigated the functional role of *TRIB3* in lipid metabolism *in vitro* by transfecting YMECs with an overexpression vector and an interference vector. The results showed that after overexpression of *TRIB3*, the mRNA expression levels of 10 genes related to milk fat metabolism in YMECs were very significantly downregulated (*p* < 0.01), the total triglyceride content was very significantly decreased (*p* < 0.001), lipid droplet accumulation was reduced, and the protein expression level of *p*-Akt was very significantly decreased (*p* < 0.01). The downregulation of the above protein levels was weakened by the addition of the Akt phosphorylation agonist SC76. After interference with *TRIB3*, the expression levels of 9 genes related to milk fat metabolism in YMECs were very significantly upregulated (*p* < 0.01), the total triglyceride content was very significantly increased (*p* < 0.01), lipid droplet accumulation was increased, and the protein expression levels of p-Akt (*p* < 0.01), PPARg (*p* < 0.05) and p-mTOR (*p* < 0.01) were very significantly upregulated. The upregulation of the above protein levels was reversed by the addition the Akt phosphorylation inhibitor MK2206. These results indicated that the *TRIB3* gene negatively regulates lipid synthesis in YMECs. *TRIB3* affects lipid metabolism by inhibiting Akt phosphorylation, which lays a theoretical foundation for further studies on lipid metabolism in YMECs and for improving yak milk quality.

## 1 Introduction

The fat content of yak milk ranges from 5.5 to 7.2%, approximately twice that of regular milk (1). Furthermore, yak milk exhibits a high concentration of functional fatty acids (2). Eicosapentaenoic acid (EPA) and docosahexaenoic acid (DHA) are unique nutritional components present in yak milk (3). Lipids constitute the primary energy source in milk, accounting for about 40–55% of its total energy content (4). Triglycerides (TAGs) represent the predominant component of milk fat, comprising 95–98% of the overall composition, followed by diacylglycerol, phospholipids, cholesterol, and other bioactive substances (5). Fatty acids serve as crucial building blocks for triglyceride synthesis; their composition in milk is influenced by both internal factors such as individual genetic characteristics and physiological conditions, as well as external factors including feeding management and environmental conditions (6). Fatty acids in milk originate from two main sources: direct absorption from blood-derived fatty acids and synthesis from precursor substances within mammary epithelial cells (7). The process of fat synthesis and secretion in mammary epithelial cells encompasses four stages: *de novo* fatty acid synthesis, triglyceride formation, droplet assembly, and subsequent packaging into secretory vesicles before release (8). These processes involve several key genes and transcriptional regulators responsible for regulating milk fat synthesis (9), whereby alterations in their expression can lead to changes in this biological process.

The Tribbles (*TRIBs*) gene is a member of the serine/threonine protein kinase-like protein gene family, which was first discovered in Drosophila, and is a nuclear gene that induces apoptosis and inhibits mitosis (10). Members of the Tribbles family belong to pseudokinase proteins, and a total of three homologues have been identified in mammals, including *TRIB1, TRIB2* and *TRIB3*, all of which contain a serine/threonine protein kinase-like domain (11). *TRIB3* plays a regulatory role in several signal transduction pathways (12). It can regulate protein activity by promoting the degradation and ubiquitination of other proteins; therefore, it functions as a regulatory protein (13). Many studies have shown that the *TRIB3* gene is also involved in the regulation of glucose metabolism in the liver (14) and skeletal muscle (15). In addition, it is involved in lipid metabolism (16). *TRIB3* mainly acts on cellular metabolic process by strongly inhibiting Akt phosphorylation, thereby affecting the insulin signaling pathway and inhibiting lipid metabolism, glucose metabolism, and other key functional mechanisms (17). *TRIB3* has been shown to inhibit Akt phosphorylation by binding to Akt phosphorylation sites at Thr308 and Ser473 residues. Phosphorylation of Akt affects the activity of downstream targets, including the Thr308 phosphorylation sites regulated by the mTOR complex 2 (mTORC2) and 3-phosphoinositol-depend-ent protein kinase-1 (PDK1) (18). However, this is not the case in all cell types. Studies have shown that overexpression of *TRIB3* in lymphocytes does not inhibit phosphorylation of the Thr308 residue of Akt (19). *TRIB3* may selectively inhibit phosphorylation of Ser473 residue of Akt in renal tubular cells. There is an interaction between *TRIB3* and Akt. *TRIB3* negatively regulates the Akt signaling pathway by inhibiting the phosphorylation level of Akt.

In addition, many studies focus on the effects of *TRIB3* on genes involved in lipid metabolism. For example, Qi et al. found that acetyl-CoA carboxylase (*ACACA*) is the rate-limiting enzyme in fatty acid synthesis, and *TRIB3* can promote the degradation of *ACACA*, thereby reducing the synthesis of free fatty acids in cells, decreasing intracellular lipid levels, and further promoting lipolysis (12). Other studies have shown that *TRIB3* expression in adipocytes reduces the mRNA level of *PPARg* and intracellular triglyceride levels, whereas inhibition of TRIB3 expression by RNA interference increases the mRNA level of *PPARg* and intracellular triglyceride levels (20). In summary, the results of many studies indicate that research on *TRIB3* in lipid metabolism mainly focuses on the interaction between *TRIB3* and the key genes involved in fatty acid metabolism. At the cellular level, *TRIB3* plays a negative regulatory role in lipid metabolism, affecting the process of cellular lipid synthesis by downregulating the mRNA and protein expression of key genes involved in lipid metabolism (8).

At present, there are relatively few studies on the *TRIB3* gene in ruminants, and even fewer studies have focused on yaks. The mechanism by which *TRIB3* regulates lipid metabolism in Yak Mammary Epithelial Cells (YMECs) remains unclear. Therefore, in this study, YMECs were used as experimental materials to analyze the effect of the *TRIB3* gene on milk fat metabolism by constructing *TRIB3* interference and overexpression vectors and applying gene overexpression and interference techniques. By overexpressing and interfering with the *TRIB3* gene in YMECs, the effect of *TRIB3* on genes related to yak milk fat metabolism were analyzed, including three involved in *de novo* fatty acid synthesis, namely acetyl-CoA carboxylase (*ACACA*) (21), fatty acid synthase (*FASN*) (22) and stearoyl-Coa desaturase 1 (*SCD1*) (23). Six genes related to fatty acid uptake and transport were also analyzed: acyl-CoA synthetase short-chain family member 2 (*ACSS2*) (24), fatty acid-binding protein 3 (*FABP3*) (25), fatty acid-binding protein 4 (*FABP4*) (26), acyl-CoA synthetase long-chain family member 1 (*ACSL1*) (27), acyl-CoA synthetase long-chain family member 4 (*ACSL4*) (28) and lipoprotein lipase (*LPL*) (29). In addition, three genes related to triglyceride synthesis and lipid droplet accumulation were analyzed: glycerol-3-phosphate acyltransferase mitochondrial (*GPAM*) (27), diacylglycerol acyltransferase 1 (*DGAT1*) (30), and perilipin 1 (*PLIN1*) (27). Three fatty acid-related transcription factors are peroxisome proliferator-activated protein (*PPARg*) (31), sterol regulatory element-binding protein cleavage-activating protein (*SCAP*) (32) and insulin-induced gene 1 (*INSIG1*) (33), and its effects on the expression levels of AKT, mTOR and other proteins, total triglyceride content and lipid drop accumulation in YMECs. To provide theoretical basis for elucidating the regulation of milk fat metabolism by *TRIB3* gene in mammary epithelial cells of yaks.

## 2 Materials and methods

### 2.1 Collection of experimental animals and tissue specimens

Three healthy yaks in their second lactation at 70 days in milk (DIM) were selected from a slaughterhouse in Qinghai Province, China. Mammary gland tissues were collected immediately after slaughter and rapidly frozen in liquid nitrogen for subsequent analysis. Primary yak mammary epithelial cells (YMECs) used in this study were previously isolated and cultured in our laboratory according to the method described in a previous study (34). The cloning, sequencing, and sequence analysis of the *TRIB3* gene have been completed in our earlier work and were reported previously (35).

### 2.2 Construction of the overexpression vector pcDNA3.1-TRIB3

According to the cloning and sequencing results of the yak *TRIB3* gene, a pair of specific primers containing Hind III and Xba I restriction sites was designed using Oligo 7 software. The sequencing of the upstream and downstream primers were as follows:

F: CCCAAGCTTATGCGAGCAAGCCCCC;

R: GCTCTAGATCAGCCATAGAGACCCATATCTCTTTCT. (Gene ID:102288142). The PCR reaction system consisted of 1 μL of 10 μM upstream primer, 2 μL of pMD19-T-TRIB3 plasmid, 10 μL of 2 × Primer Star MAX enzyme, and 11 μL of sterilized ddH_2_O in a total volume of 20 μL. The reaction conditions were as follows: predenaturation at 98 °C for 3 min; (98 °C for 10s, 61 °C for 5 s, 72 °C for 10s) × 30 cycles; 72 °C for 3 min; The samples were stored at 4 °C. At the end of the PCR reaction, the PCR products were examined by running gel on 1% agarose gel electrophoresis.

At the end of electrophoresis, the gel with the target size of bands was cut out, and the target bands with restriction sites at both ends were recovered by DNA purification and recovery kit (EG101-01, full gold). The empty plasmid was extracted from the bacterial solution of pcDNA3.1 (+) plasmid (V79020, Invitrogen) stored in the laboratory using the High purity plasmid medium kit (EM101-01, full Gold) after amplification. The eukaryotic expression vector pcDNA3.1 (+) and purified PCR products were then digested with Hind III (1060S, TAKARA) and Xba I (1093S, TAKARA) restriction enzymes for 2 h at 37 °C.

T4 DNA ligase was used to link the target band with the pcDNA3.1 empty vector to construct the pcDNA3.1-TRIB3 overexpression vector. The connected pcDNA3.1-TRIB plasmid was transformed into DH5α *E. coli* competent cells (9,057, TAKARA). Because the culture medium was Amp resistant, monoclonal cells could be picked out in solid culture dishes, and then cultured with liquid LB until the bacterial solution was turbidized for bacterial solution detection. The positive plasmid was sent to Wuhan Aoke Dingsheng Biotechnology Co., LTD for sequencing, and the pcDNA3.1-TRIB overexpression vector was obtained. The sequencing work in this test was carried out by the company.

### 2.3 Preparation of shRNA interference vectors

shRNA sequence design: four corresponding interference fragments were designed according to the *TRIB3* gene sequence, and then the designed fragments were sent to Shanghai Sangon Biological Company for synthesis. The interfering fragments designed for different sites of the *TRIB3* gene are shown in Table 1. The interference vector pSliencer3.0-H1 was prepared by EcoR I and BamH I, and the products after double enzyme digestion were detected and observed by electrophoresis, and then the target band was cut and recovered. The Oligo fragments were synthesized and then dissolved and diluted in ddH_2_O. Then 4 μL of upper and downstream primers and 2 μL of annealing buffer (5×) were added to a clean centrifuge tube and put into a PCR instrument to perform the following procedure: 95 °C,5 min; 80 °C,4 min; 75 °C,4 min; 70 °C,4 min; 65 °C,2 min; 60 °C,2 min; 55 °C,2 min; 50 °C,2 min; 45 °C,2 min; 40 °C,2 min; 37 °C,2 min; 20 °C,20 min.

**Table 1:** shRNA sequences of TRIB3.

Serial number	Sequence
TRIB3-82-F	GATCCGAGTGTCCCAGCCAGAAACAACTCGAGTTGTTTCTGGCTGGGACACTCTTTTTG
TRIB3-82-R	AATTCAAAAAGAGTGTCCCAGCCAGAAACAACTCGAGTTGTTTCTGGCTGGGACACTCG
TRIB3-196-F	GATCCTCCAGGCTTGGACCCTATGTTCTCGAGAACATAGGGTCCAAGCCTGGATTTTTG
TRIB3-196-R	AATTCAAAAATCCAGGCTTGGACCCTATGTTCTCGAGAACATAGGGTCCAAGCCTGGAG
TRIB3-223-F	GATCCCTGGGAACACAGCAGTGAATACTCGAGTATTCACTGCTGTGTTCCCAGTTTTTG
TRIB3-223-R	AATTCAAAAACTGGGAACACAGCAGTGAATACTCGAGTATTCACTGCTGTGTTCCCAGG
TRIB3-1038-F	GATCCGGAGGAAGGAGAAAGAGATATCTCGAGATATCTCTTTCTCCTTCCTCCTTTTTG
TRIB3-1038-R	AATTCAAAAAGGAGGAAGGAGAAAGAGATATCTCGAGATATCTCTTTCTCCTTCCTCCG
shRNA-NC	GATCCCAACAAGATGAAGAGCACCAACTCGAGTTGGTGCTCTTCATCTTGTTGTTTTTG
shRNA-NC	AATTCAAAAACAACAAGATGAAGAGCACCAACTCGAGTTGGTGCTCTTCATCTTGTTGG

^1^F: Forward primer; R, Reverse primers; NC, Negative control.

The mixture after annealing was diluted 10 times, and the following components were added to the centrifuge tube: 10 × buffer 1 μL, T4 DNA ligase 0.1 μL, annealing mixture 1 μL, 13 μL carrier recovered after enzyme digestion, and ddH_2_O was supplemented to 20 μL. After mixing and centrifugation, the reaction was carried out for 30 min at 22 °C on a PCR instrument.

The steps of transformation of the ligation product into DH5α, overnight culture in LB solid medium, selection of monoclonal antibodies and shaking bacteria, extraction of plasmid and sequencing can be referred to as the related steps of construction of overexpression vector.

### 2.4 Resuscitation and transfection of yak mammary epithelial cells

The cryopreserved primary yak mammary epithelial cells were taken out and immediately thawed rapidly in a 37 °C water bath. After appropriate disinfection and washing, the cells were transferred to a biosafety cabinet. An appropriate amount of culture medium was added, followed by centrifugation. The cell pellet was resuspended and seeded into cell culture flasks. Cell density and growth status were observed under a microscope, and flasks with higher cell density were subcultured at an appropriate ratio. All cells were then incubated in a CO₂ constant-temperature incubator, and the culture medium was replaced regularly. When appropriate, the cells were passaged, and according to the experimental requirements, a certain number of cells were seeded into 6-well plates for further culture. When the cell confluence in the 6-well plates reached 80–90%, drug treatment experiments were performed.

During the experiment, the Akt phosphorylation activator SC76 (3 μg/mL) or the Akt phosphorylation inhibitor MK2206 (5 μM) was added to the cell culture system and incubated for 24 h for subsequent experiments. Meanwhile, plasmid transfection was performed using Lipofectamine 2000 according to the manufacturer’s instructions. After transfection, the cells were cultured for 36 h and subjected to Oil Red O staining. After 48 h of culture, total RNA was extracted for real-time quantitative PCR (RT-qPCR) analysis and triglyceride measurement.

In addition, 24 h after transfection, SC76 (3 μg/mL) or MK2206 (5 μM) was added to the culture system and the cells were further treated for 24 h. After treatment, the cells were collected for subsequent Western blot analysis and lipid metabolism–related experiments.

### 2.5 Reverse transcription quantitative polymerase chain reaction

Forty-eight hours after transfection, the cells were digested and collected to extract RNA (15,596,018, Life Technologies), reverse transcribed into cDNA by reverse transcription kit (KR118, Tiangen), and then the mRNA expression changes of lipid metabolism-related genes were detected by RT-qPCR. A total of 15 lipid metabolism-related genes were selected, including 3 *de novo* fatty acid synthesis genes (*ACACA, FASN* and *SCD1*). Six fatty acid uptake and transport genes, including *ACSS2, FABP3, FABP4, ACSL1, ACSL4* and *LPL;* Three genes related to triglyceride synthesis and lipid droplet accumulation, namely *GPAM, DGAT1* and *LPIN1*, and three fatty acid related transcription factors, namely *PPARg, SCAP* and *INSIG1*. *β*-actin was selected as the internal reference gene, and the primers of each gene were designed and sent to Shanghai Sangon Biological Company for synthesis. The specific primer sequences are shown in Table 2.

**Table 2:** Primer sequence information for RT-qPCR.

Gene	NCBI Accession ID	Primer sequence (5’-3’)	Tm (°C)	Product length (bp)
*ACACA*	XM_005888164.2	F: CCAGCAAAACAAAGCTACCCTG R: CAAACTTATCCCTTGCTCGGAA	60	158
*FASN*	XM_005905364.2	F: CTGAAGAACATCCTGGCCGAT R: CACCAGGACGCACCGAATCCG	60	138
*FABP3*	XM_005906169.2	F: CTGTCCCTTCAGCTAAGCCTA R: CCAGAGCCACAGTATTACGAG	60	109
*SCD*	XM_005892055.2	F: TTATCCGACCTAAGAGCCGAGA R: CACAGATACCACGGCACGAG	60	110
*LPL*	XM_005902304.2	F: GAACTGGATGGCGGATGAAT R: AAGCCGGTTATCCTGTTGAC	60	127
*ACSL1*	XM_005906554.2	F: GGCACCTTGAAGATCATCGAC R: CCTGAGCGATAGGTTCACTCC	60	115
*FABP4*	XM_005897260.2	F: AACCCACTTTGATCATCAGT R: GCACCTTCATCTAAGTTTACGAT	60	160
*ACSL4*	XM_005905770.2	F: AGAAAAAGAGAAAATCTAAAAACGC R: TCTGCTCCAGGGATGTCTAT	60	133
*ACSS2*	XM_005900428.2	F: GGCTCTGCACTCCATTGTGT R: GCCAGCTCCTTCAGGTTGACA	60	138
*GPAM*	XM_005906833.2	F: CACCAATACGATCCCGGACA R: GATAACGCCTCTTGCTATGCT	60	139
*DGAT1*	XM_014478833.1	F: CCCTACTCCAAGCCCATCACC R: CTTCACCAGGGCTTGCACGTA	60	120
*LPIN1*	XM_005903204.2	F: CTTCAGATGCGTTTAGTGACC R: CCTCGACATCGTCTTCGTTC	60	120
*PPARg*	XM_005902846.1	F: AGAGTACGCCAAGAATATCCC R: AGCATCGTGTAAATGATCTCG	60	99
*INSIG1*	XM_014481357.1	F: TGGGCATCACCATCGCCTT R: TCCTATGTTCCCCACCGTCAC	60	152
*SCAP*	XM_014483313.1	F: ACGGGCATCAAGCTCTACTCC R: TCCCCGTAGTTCAGGTCCCAA	60	128
*β-actin*	XM_005897464.1	F: TGTTAGCTGCGTTACACCCT R: CTGTCACCTTCACCGTTCC	60	168

^1^F: Forward primer; R: Reverse primers.

According to the instructions of the fluorescence quantitative kit (FP205, Tianguan), a 20 μL reaction system was selected to detect the mRNA expression of the above genes. The reaction program was 95 °C for 15 min; (95 °C for 10 s, 60 °C for 20 s, and 72 °C for 20 s) 40 cycles.

### 2.6 Protein extraction and Western blotting tests

YMECs were seeded in 6-well plates and cultured until the cell confluence reached 70–80%, at which point plasmid transfection was performed. According to the experimental design, three wells in the 6-well plate were transfected with the overexpression plasmid pcDNA3.1-TRIB3, while the remaining three wells were transfected with the empty control plasmid pcDNA3.1-NC. Meanwhile, liposome-mediated transfection was used to introduce shRNA-NC and shRNA-196 plasmids, respectively, to perform gene interference experiments.

During the transfection process, serum-free medium was used. After adding the transfection complexes, the cells were further incubated for 5–6 h. The transfection medium was then discarded and replaced with normal complete culture medium for continued incubation. After 72 h of transfection, the cells were digested with trypsin, collected, and centrifuged, and the supernatant was discarded. Total cellular proteins were extracted according to the instructions of the total protein extraction kit, and the protein concentration was determined. After normalizing the protein concentration, the samples were denatured and subsequently used for Western blot analysis.

The primary antibody used was anti-TRIB3 (66,702, Proteintech), and the secondary antibody was HRP-conjugated goat anti-rabbit IgG (Bioss, China).

### 2.7 Measurement of triglycerides

Transfection of overexpression and interference plasmids was performed in 6-well plates. Three Wells were experimental group, transfected with overexpression plasmid or interference plasmid; The remaining 3 Wells were used as control and transfected with pcDNA3.1-NC or shRNA-NC empty plasmid. After transfection, the cells were cultured for another 48 h, and the cells were washed 3 times with PBS. Then the experimental operation was carried out according to the instructions given by the triglyceride detection kit (E1013, Prielei) and the concentration of TAG in the cells was determined.

### 2.8 Oil red O staining

Experimental (*n* = 3) and control (*n* = 3) groups were set up in 12-well plates and subsequently stained using oil red O. The specific operation is as follows:

(1) Cell slides were made in 12-well plates. Collagen solution with a concentration of 2 mg/mL was prepared with PBS, and the slides were treated in collagen solution in advance to promote cell adhesion and enhance its adhesion to the slides.1 mL collagen solution was added to each well and incubated for 30 min at 37 °C. The collagen solution was aspirated and washed several times with PBS.
(2) Cells were seeded in 12-well plates with cell slides and transfected accordingly. After 36 h of culture, an appropriate amount of PBS buffer was added to each well to clean the cells.4% paraformaldehyde was added and fixed at room temperature for 30 min, and then washed with PBS for more than 10 min, during which multiple fluid exchanges were required.
(3) An appropriate amount of 60% isopropanol was added to 12-well plates and then immersed for 1–2 min.
(4) Add 1 mL working solution to each well and dye for 10 min. (Preparation of oil red O working solution: add 0.5 g of oil red dry powder to 100 mL of isopropanol to fully dissolve it. Mix the liquid and ddH_2_O at a volume ratio of 3:2, stand for 10 min, filter with filter paper, and the working liquid needs to be mixed on the spot).
(5) Add 60% isopropanol for 2 to 3 min, and rinse with pure water.
(6) Add lapis lazuli blue staining solution for 3 min, and rinse with pure water.
(7) Hematoxylin was used for nucleus staining for 3–10 min, then rinsed with pure water, 1% hydrochloric acid was added to differentiate, rinsed with pure water, 1% ammonia was added to turn blue, and rinsed with pure water.
(8) Remove the cell slide and seal it with glycerin gelatin. They were observed under a microscope, photographed and recorded, and oil-red O-stained pictures were collected.

### 2.9 Statistical analysis

Data were analysed using SPSS software (Ver.17.0, SPSS Inc., Chicago, IL, United States). One-way ANOVA was used to test whether significant differences existed. Values in the figures are given as the means±SEM (standard error of the mean). Differences among multiple groups were analyzed using one-way analysis of variance (ANOVA) followed by Duncan’s multiple range test. Differences were considered statistically significant at *p* < 0.05. Different letters in the figures indicate significant differences among groups.

## 3 Results

### 3.1 Overexpression and interference efficiency of TRIB3 gene in mammary epithelial cells

**Figure 1:**
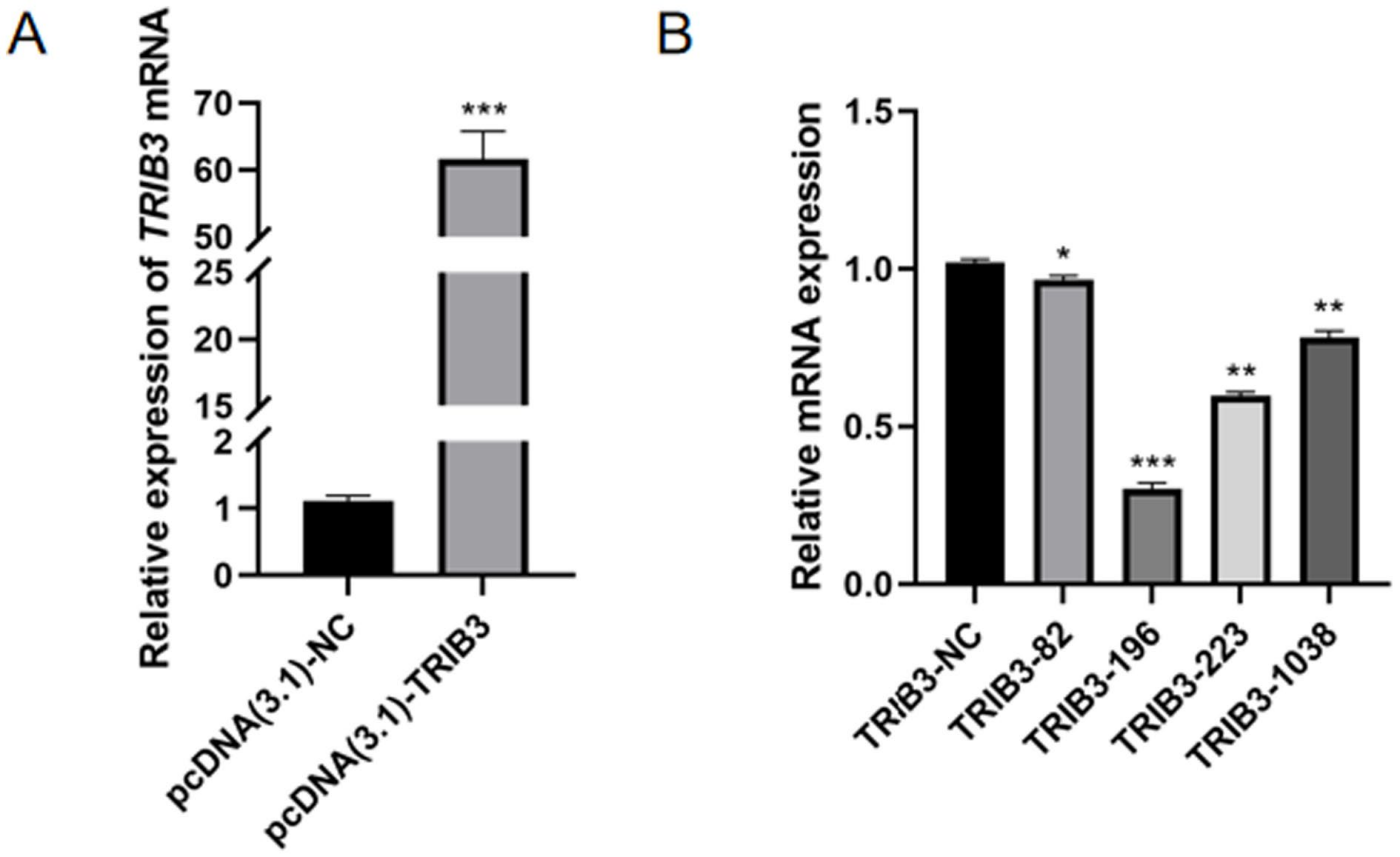
TRIB3 overexpression and interference efficiency in YMECs. **(A)** TRIB3 overexpression efficiency in YMEC. **(B)** TRIB3 interference efficiency in YMECs. pcDNA(3.1)-NC: negative control group; pcDNA(3.1)-TRIB3: TRIB3 overexpression group. shTRIB3-NC: negative control group; TRIB3-82/196/ 223/1038: TRIB3 interference group.*** indicates very extremely significant differences (*p* < 0.001);** indicates very significant difference (*p* < 0.01);* indicates significant differences (*p* < 0.05).

The overexpression plasmid and its control group and different interference plasmids and their control group were transfected into YMECs, respectively. After 48 h of normal culture, the overexpression and interference efficiency of the vector were detected by RT-qPCR to detect the mRNA expression of the TRIB3 gene, and the results are shown in Figure 1. The results showed that the expression of TRIB3 mRNA in YMECs transfected with pcDNA3.1-TRIB3 overexpression plasmid was very extremely significantly higher than that in YMECs transfected with pcDNA3.1 empty plasmid (*p* < 0.001; Figure 1A). After overexpression, the expression of TRIB3 mRNA increased by more than 60 times, which could meet the requirements of subsequent experiments and could be used for subsequent experiments. Compared with the negative control group, the TRIB3-196 plasmid showed the best interference effect on *TRIB3* and very extremely significantly reduced the expression of TRIB3 mRNA (*p* < 0.001; Figure 1B). Therefore, we selected the interference vector shRNA-196 as the material for the subsequent interference experiment.

### 3.2 Effect of *TRIB3* overexpression and interference on total triglyceride content in YMECs

**Figure 2:**
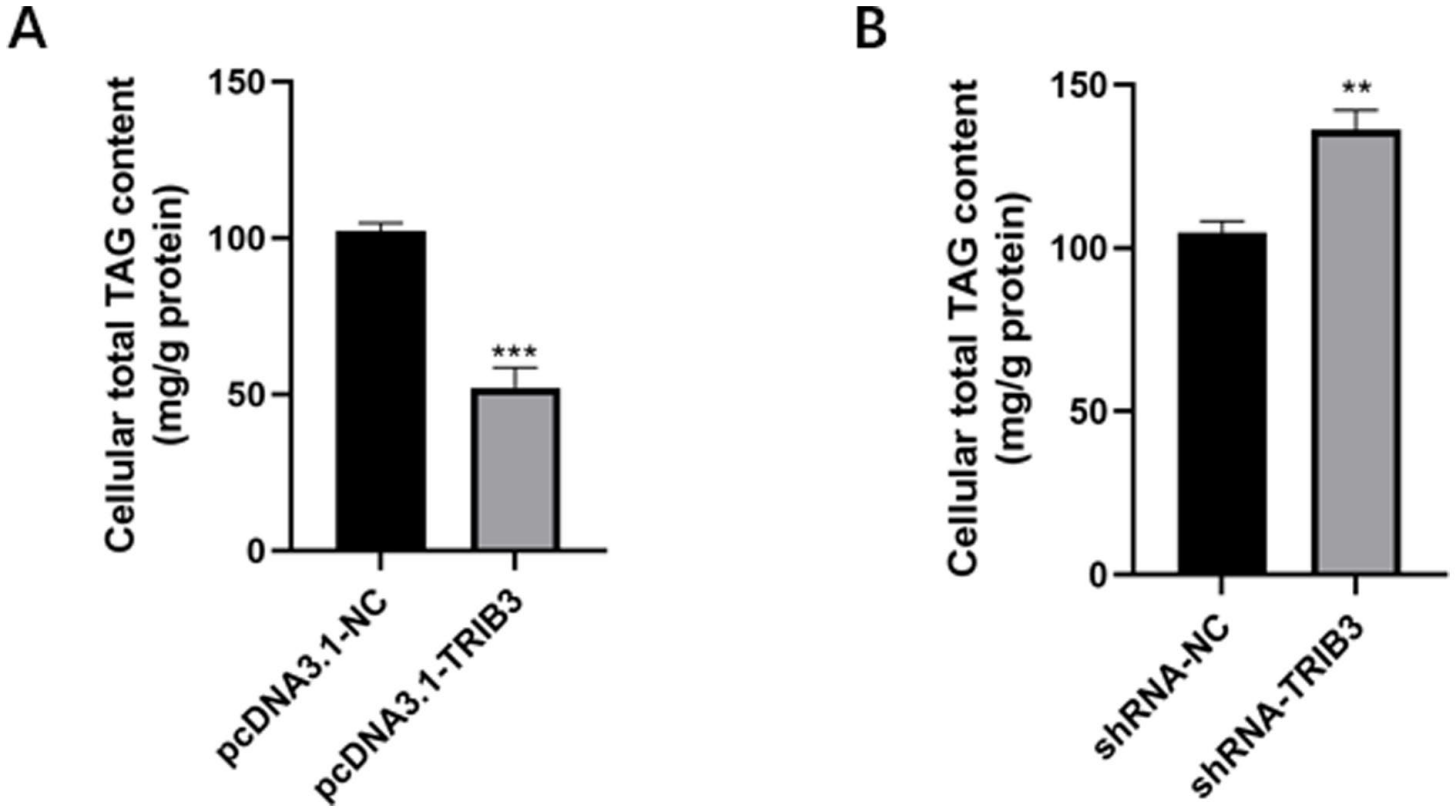
**(A)** Effects of overexpression of TRIB3 gene on total triglyceride content in YMECs. **(B)** Effects of interfering of TRIB3 gene on total triglyceride content in YMECs. *** indicates very extremely significant differences (*p* < 0.001); ** indicates very significant difference (*p* < 0.01).

The overexpression plasmid and its control group, and the interference plasmid and its control group were transfected into YMECs by liposome transfection technology, respectively. After 48 h of culture, the total triglyceride of YMECs was extracted and detected. The results showed that the overexpression of the *TRIB3* gene significantly decreased the total triglyceride content (*p* < 0.001; Figure 2A), indicating that the synthesis of triglycerides was reduced, that is, the synthesis of lipids within YMECs was inhibited. However, the knockdown of *TRIB3* significantly increased the content of total triglyceride (*p* < 0.01; Figure 2B), indicating increased triglyceride synthesis, that is, enhanced lipid synthesis within YMECs.

### 3.3 Effect of *TRIB3* overexpression and interference on lipid droplet accumulation in YMECs

**Figure 3:**
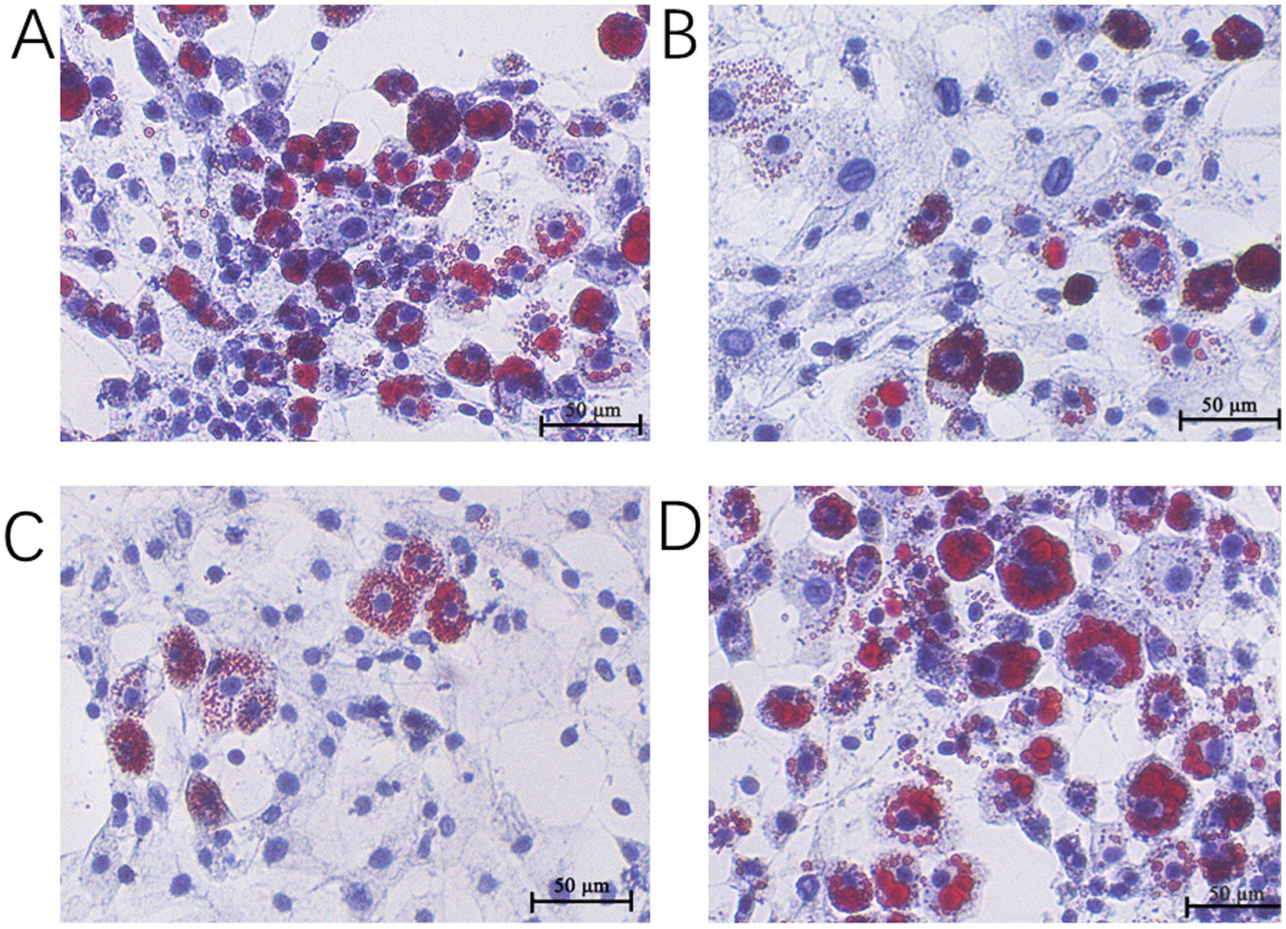
Effect of overexpressed and interfering of TRIB3 gene on lipid droplet accumulation in YMECs (400×). **(A)** TRIB3 overexpression group. **(B)** Negative control group. **(C)** TRIB3 interference group. **(D)** Negative control group.

The overexpression plasmid and its control group, and the interference plasmid and its control group were transfected into YMECs by liposome transfection technology, and oil red O staining was performed after 36 h of culture. The results showed that after the overexpression of the *TRIB3* gene, the formation of lipid droplets in YMECs was reduced, and the accumulation of lipid droplets was significantly reduced (Figures 3A,B). In other words, the overexpression of *TRIB3* inhibited the formation of lipid droplets in YMECs, and the accumulation of lipid droplets was significantly reduced. However, after interfering with the *TRIB3* gene, the formation of lipid droplets in YMECs increased, and the lipid droplets became larger and accumulated significantly (Figures 3C,D), indicating that interfering with *TRIB3* could significantly increase the accumulation of lipid droplets in YMECs.

### 3.4 The mRNA expression changes of lipid metabolism-related genes in YMECs after overexpression and interference of *TRIB3* gene

**Figure 4:**
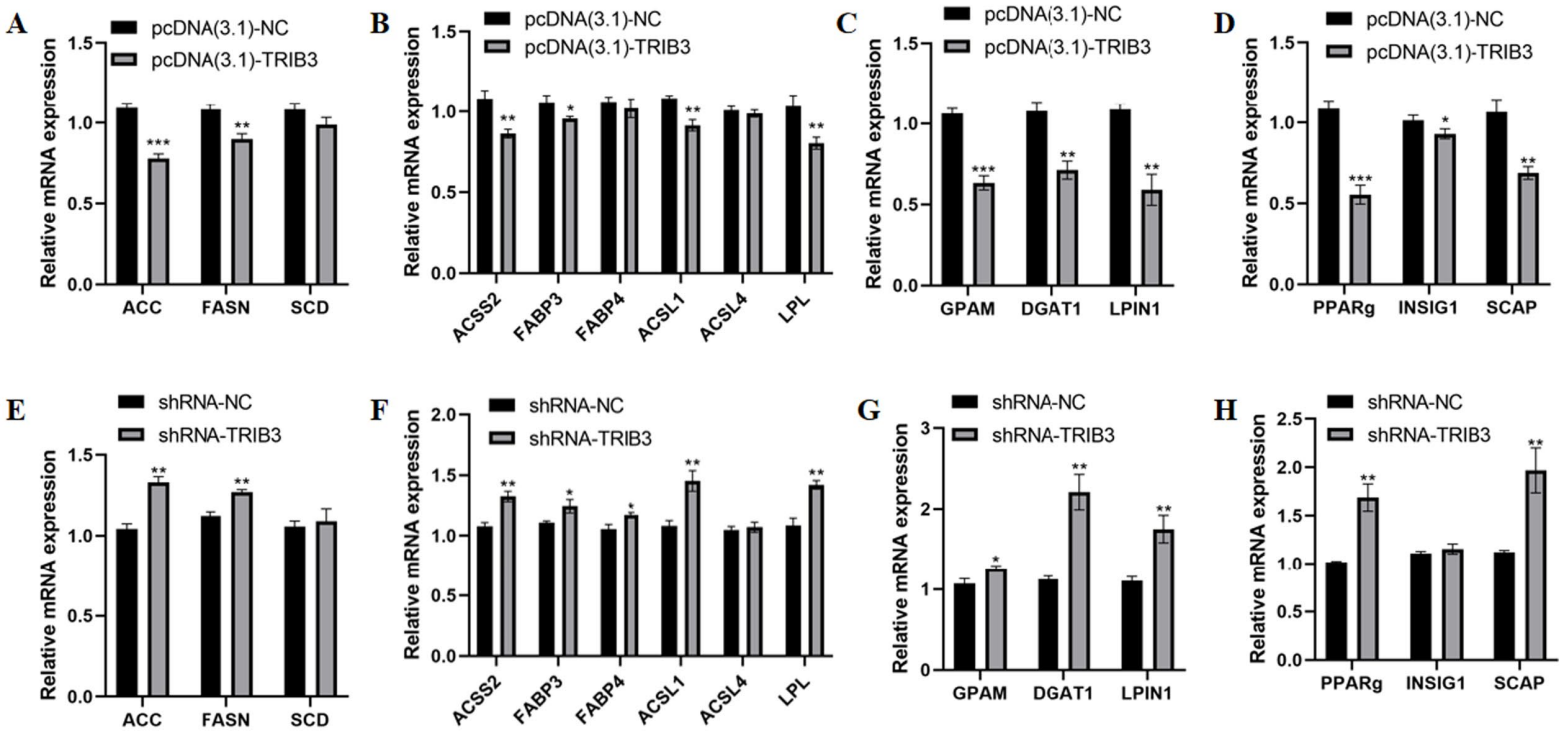
Effects of overexpression and interfering of TRIB3 gene on mRNA expression of 15 lipid metabolism-related genes in YMECs. **(A,E)** mRNA expression of genes related to *de novo* fatty acid synthesis (ACACA, FASN, SCD1). **(B,F)** mRNA expression of genes related to uptake and activation (ACSS2, FABP3, FABP4, ACSL1, ACSL4, LPL). **(C,G)** mRNA expression of genes related to triglyceride synthesis (GPAM, DGAT1, LPIN1). **(D,H)** mRNA expression of genes related to transcription factors (PPARg, INSIG1, SCAP). pcDNA(3.1)-NC:negative control group; pcDNA(3.1)-TRIB3: TRIB3 overexpression group.*** indicates very extremely significant differences (*p* < 0.001);** indicates very significant difference (*p* < 0.01);* indicates significant differences (*p* < 0.05).

The overexpression plasmid and its control group, and the interference plasmid and its control group were transfected into YMECs, respectively. After 48 h of normal culture, the cells were digested and collected. RNA was extracted and reverse-transcribed into cDNA. RT-qPCR was used to detect the mRNA expression levels of 15 genes related to lipid metabolism. The results showed that the mRNA expression levels of *ACACA* (Figure 4A), *GPAM* (Figure 4C) and *PPARg* (Figure 4D) were very extremely significantly downregulated after *TRIB3* gene overexpression (*p* < 0.001), the expression levels of *FASN* (Figure 4A), *ACSS2* (Figure 4B), *ACSL1* (Figure 4B), *LPL* (Figure 4B), *DGAT1* (Figure 4C), *LPIN1* (Figure 4C), and *SCAP* (Figure 4D) were very significantly downregulated(*p* < 0.01), and the expression levels of *FABP3* (Figure 4B) and *INSIG1* (Figure 4D) were significantly downregulated (*p* < 0.05), but the expression of *SCD* (Figure 4A), *FABP4* (Figure 4B), and *ACSL4* (Figure 4B) did not change significantly.

The expression levels of *ACACA* (Figure 4E), *FASN* (Figure 4E), *ACSS2* (Figure 4F), *ACSL1* (Figure 4F), *LPL* (Figure 4F), *DGAT1* (Figure 4G), *LPIN1* (Figure 4G), *PPARg* (Figure 4H) and *SCAP* (Figure 4H) were very significantly upregulated after *TRIB3* gene interference (p < 0.01), and the expression levels of *FABP3* (Figure 4F), *FABP4* (Figure 4F), and *GPAM* (Figure 4G) genes were significantly upregulated (*p* < 0.05), but there was no significant change in the expression of *SCD* (Figure 4E), *ACSL4* (Figure 4F) and *INSIG1* (Figure 4H).

### 3.5 Effects of overexpression and interference of *TRIB3* on the expression levels of p-Akt, T-Akt, PPARg and other proteins in YMECs

To investigate whether *TRIB3* regulates lipid metabolism through the Akt signaling pathway, the protein expression levels of TRIB3, Akt, p-Akt, and PPARg were detected by Western blot analysis.

**Figure 5:**
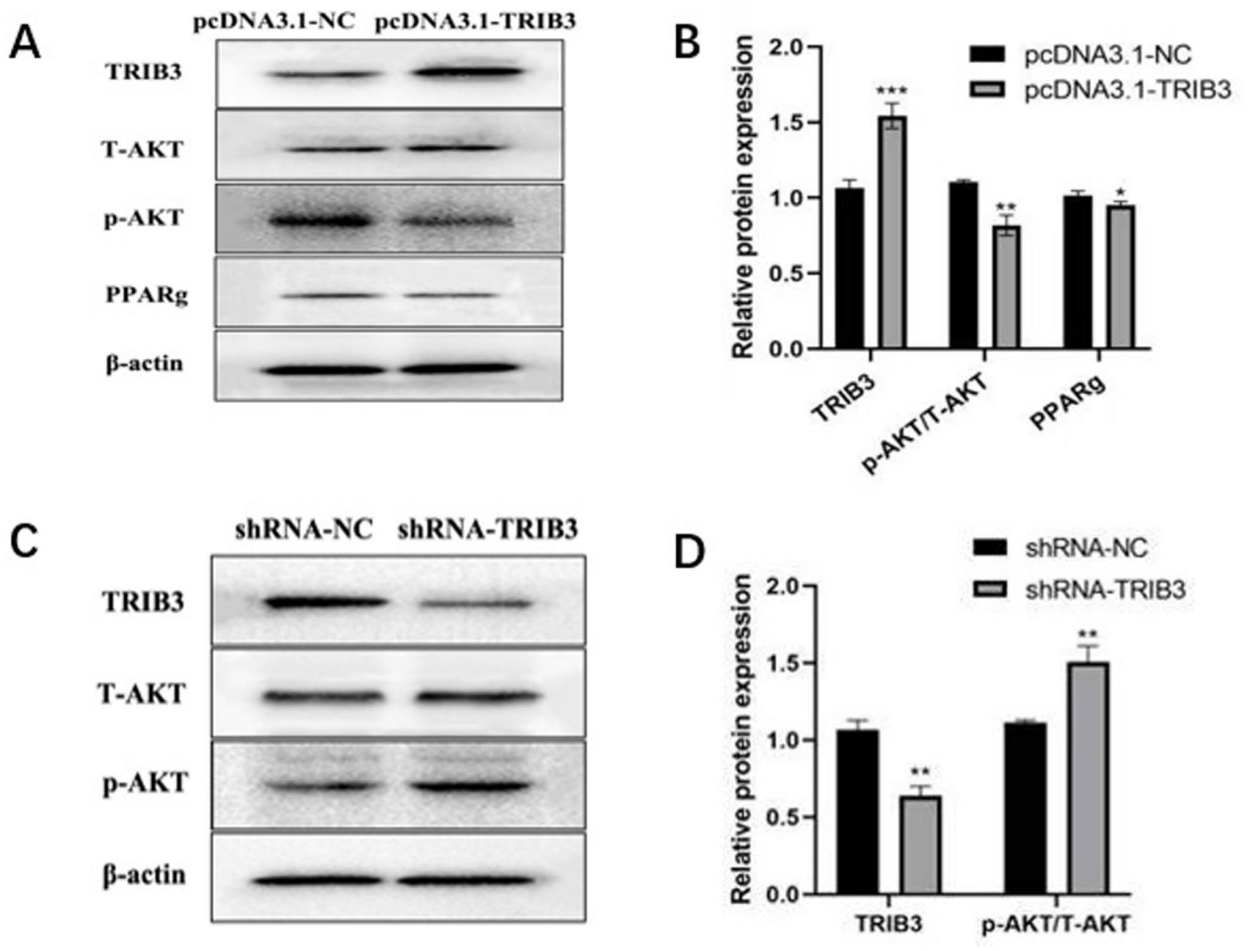
**(A,B)** Effect overexpression of TRIB3 on the expression of related proteins (TRIB3, T-Akt, p-Akt, PPARg). **(C,D)** Effect interference of TRIB3 on the expression of related proteins (TRIB3, T-Akt, p-Akt). *** indicates very extremely significant differences (*p* < 0.001);** indicates very significant difference (*p* < 0.01);* indicates significant differences (*p* < 0.05).

As shown in Figures 5A,B, after overexpression of *TRIB3* in YMECs, the protein expression level of p-Akt was significantly decreased compared with the control group, whereas the protein expression level of total Akt (T-Akt) showed no significant change. Meanwhile, the protein expression level of PPARg was also decreased.

After interference with *TRIB3*, the protein expression level of p-Akt was increased compared with the control group, while the expression level of T-Akt remained unchanged (Figures 5C,D). In addition, the protein expression levels of PPARg and p-mTOR were also increased after *TRIB3* interference, indicating that *TRIB3* negatively regulates Akt phosphorylation and affects downstream signaling molecules.

**Figure 6:**
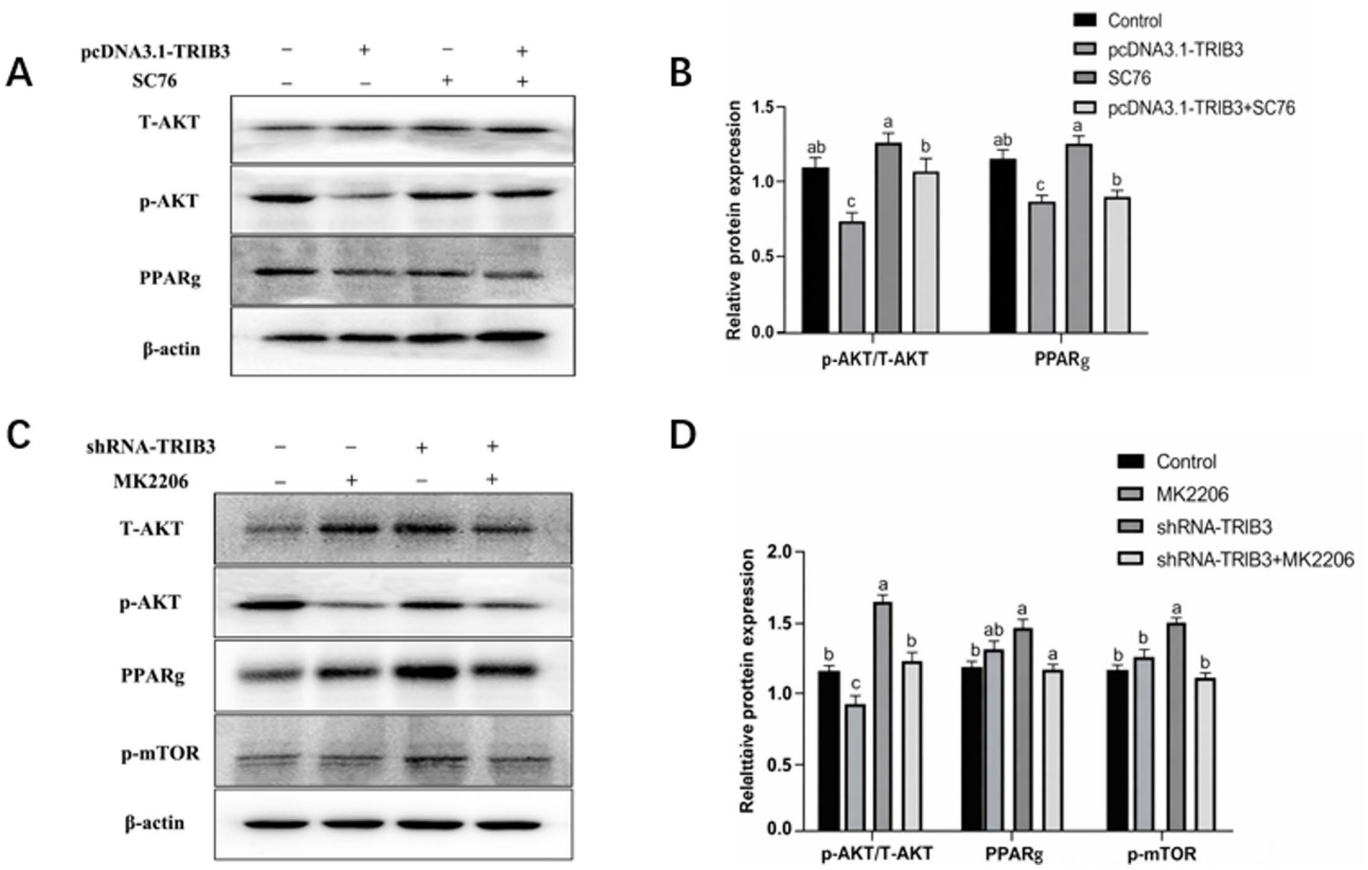
**(A,B)** Effect overexpression of TRIB3 with the co treatment and SC76 on the expression of related proteins (T-Akt, p-Akt, PPARg). **(C,D)** Effect interference of TRIB3 with the co treatment and MK2206 on the expression of related proteins (T-Akt, p-Akt, PPARg, p-mTOR). Diferent letters indicate significant differences among groups (*p* < 0.05).

To further verify the relationship between *TRIB3* and Akt phosphorylation, an Akt phosphorylation agonist (SC76) and inhibitor (MK2206) were used. As shown in Figures 6A,B, treatment with SC76 increased the protein expression level of p-Akt and PPARg in YMECs. However, when *TRIB3* was overexpressed together with SC76 treatment, the increase in p-Akt and PPARg protein expression was weakened, suggesting that *TRIB3* can inhibit Akt phosphorylation.

As shown in Figures 6C,D, treatment with the Akt phosphorylation inhibitor MK2206 reduced the protein expression level of p-Akt. When *TRIB3* interference was combined with MK2206 treatment, the increase in p-Akt induced by *TRIB3* interference was attenuated, and the expression levels of PPARg and p-mTOR also showed a decreasing trend.

These results indicate that *TRIB3* can regulate the Akt signaling pathway by inhibiting Akt phosphorylation and thereby affect the expression of downstream proteins such as PPARg and mTOR.

## 4 Discussion

In recent years, yak milk has been a kind of pollution-free green food for people living in the plateau environment, which is loved by more and more people (36). It is a raw material milk with great development and utilization value, so researchers have conducted more and more studies on yak milk (37) Triglyceride is the main form of milk fat (38). The composition and content of milk fat are of great significance to the nutritional value and flavor of milk products (39). Fatty acids are absorbed or synthesized in mammary epithelial cells and bound to the glycerol skeleton through esterification to form triglycerides, which are then secreted into the extracellular space in the form of lipid droplets (40). Therefore, the triglyceride level of mammary epithelial cells can reflect the lipid content in milk to a certain extent (41). In this study, the *TRIB3* gene was overexpressed and interfered in yak mammary epithelial cells. The triglyceride content was decreased after the overexpression of the *TRIB3* gene, and the triglyceride content was upregulated after the interference, indicating that the *TRIB3* gene plays a negative regulatory role in yak milk fat synthesis.

Studies show that under the regulation of *PPAR-1α*, *TRIB3* downregulates insulin signaling pathway by affecting the function of Akt (42). In addition, experiments by Salazar et al. further demonstrated that *TRIB3* inhibits Akt phosphorylation by binding to the protein kinase phosphorylation sites Thr308 and Ser473 (18). In this experiment, the expression level of p-Akt was downregulated after overexpression of *TRIB3*. After interference with *TRIB3*, the expression level of p-Akt was upregulated. The relationship between the *TRIB3* gene and Akt phosphorylation was further verified by adding Akt phosphorylation agonist SC76 and Akt phosphorylation inhibitor MK2206. This study also verifies that the inhibitory effect of *TRIB3* on Akt can upregulate the protein level of the downstream gene mTOR. In conclusion, TRIB3 may inhibit lipid metabolism in mammary epithelial cells of yaks by binding to phosphorylated form of Akt.

Both *ACACA* and *FASN* are key enzymes in the *de novo* synthesis of fatty acids during milk fat synthesis (43). *ACACA* catalyzes the conversion of acetyl coenzyme A (acetyl CoA) to malonyl-CoA. Malonyl-CoA can act as A carbon donor for fatty acid chain elongation and promote the synthesis of triglycerides and phospholipids (21). Subsequently, acetyl-CoA and malonyl-CoA are first covalently linked to the sulfhydryl group on the active group of acyl carrier protein to form acetyl-acyl carrier protein and malonyl-malonate monoacyl acyl carrier protein, which requires *FASN* to participate in this process. In addition, a large number of medium and short-chain fatty acids are produced, which can be used as raw materials for fatty acid elongation (44). In the results of studies on lipid synthesis genes in bovine mammary glands, *ACACA* mRNA was found to be upregulated to a greater extent during lactation than *FASN* (27). The results of this study indicate that *TRIB3* can regulate the expression of *ACACA* and FASN, which are related to milk fat metabolism, and then affect triglyceride accumulation. *SCD* is mainly involved in the catalysis of monounsaturated fatty acid synthesis (23). However, the results of the present study indicate that *TRIB3* cannot regulate *SCD* expression in yak mammary epithelial cells.

LPL is the main rate-limiting enzyme in the TAG metabolic pathway, and the process of endogenous fatty acid synthesis mainly relies on LPL to take up fatty acids from the blood (29). On the one hand, FABP can transport long-chain fatty acids (LCFA) generated in the body, and on the other hand, it can also combine with acyl-CoA (45). The main function of *FABP3* in the process of bovine mammary lipid synthesis is to transport fatty acids to *SCD* as raw materials in the corresponding reaction process (25). The results of this study indicate that TRIB3 can regulate the expression of LPL and *FABP3*, which are related to milk fat metabolism, and then affect triglyceride accumulation. The main function of *ACSL* is to activate fatty acids (46). It has been shown that the mRNA of *ACSL1* is dominant in the *ACSL* subtype of bovine mammary gland tissue and increases 4-fold at the onset of lactation (27). After the expression of the *ACSL4* gene is inhibited, the proliferation and differentiation efficiency of adipocytes is improved, and the accumulation of lipid droplets is also increased (28). The main function of *ACSS2* is to guide acetate into the synthesis of fatty acids, and *ACSS2* only exists in the cytoplasm (24). However, the results of the present study showed that *TRIB3* could regulate the expression of *ACSL1* and *ACSS2* genes involved in milk fat metabolism, but did not affect the expression of the *ACSL4* gene. In conclusion, *TRIB3* can regulate the expression of four fatty acid uptake and transport genes, *LPL, FABP3, ACSL1* and *ACSS*2, thereby affecting triglyceride accumulation in yak mammary epithelial cells.

GPAM and LPIN1 modify and process the intermediates of triglyceride synthesis, and they are crucial enzymes in the process of triglyceride synthesis (8). DGAT can acylate groups on diglycerides and finally synthesize triglycerides. DGAT can also directly catalyze the synthesis of triglycerides from SCFAs and medium-chain fatty acids. After triglyceride is synthesized, it aggregates to form milk fat droplets and is secreted out (47). The mRNA expression of DGAT1 is significantly increased during lactation, especially in early lactation (48). Current studies in mice have found that overexpression or interference of DGAT1 has little effect on triglyceride synthesis during lactation. However, in ruminants, DGAT1 is one of many proteins that make up the TAG synthesis pathway and plays an important role in increasing TAG in milk (49, 50). The results of this study indicate that *TRIB3* can regulate the expression of three genes involved in triglyceride synthesis and lipid droplet accumulation, namely *GPAM, LPIN1* and *DGAT1*, thereby affecting triglyceride accumulation. The change of *DGAT1* gene was consistent with previous studies.

Many studies have shown that sterol regulatory element binding protein (SREBP) plays a key role in the mammary glands of mice and cows, which can regulate milk fat synthesis accordingly (51). Both *SREBP1* and 2 are present in the ER membrane as an inactive precursor. Before entering the nucleus and activating genes containing sterolresponse elements (e.g., *ACACA*, *FASN*), they must be transported to the Golgi apparatus for activation. SCAP is an anchor protein of SREBPs, which can block the process of SREBP transport to the Golgi (52). SCAP essentially acts as a gatekeeper for the transport of inactive SREBP to the Golgi (53, 54). INSIG1 and 2 are proteins that interact with SCAP in an oxidized sterol-dependent and -independent manner and modulate the responsiveness of SREBP1 and 2 processing via SCAP, thereby altering the rate of lipogenesis (55). Experiments have shown that when *INSIG1* is overexpressed in the liver, increased INSIG1 transcript leads to decreased SREBP activity. That is, the activity of SREBP1 can be inhibited or controlled by INSIG1 during mammary lipid synthesis (56). The *PPARg* gene plays an important role in milk fat synthesis and is the centre of a network of transcriptional regulators and nuclear receptors. At present, studies have shown that there are many target genes of *PPARg*, including *ACACA, FASN, LPIN1, DGAT1, SREBP* and *INSIG1* (57). When they combine, they coordinate the activation of genes that drive the lipid synthesis mechanism and then affect the metabolic process of LCFA (27). The results of the present study indicate that *TRIB3* can regulate the expression of two fatty acid-related transcription factors, PPARg and SCAP, which in turn affect triglyceride accumulation in yak mammary epithelial cells. However, *TRIB3* could not regulate the expression of *INSIG1* gene in yak mammary epithelial cells.

Studies have shown that *TRIB3* can affect the expression of lipid metabolism-related genes. After the *TRIB3* gene is interfered with, the expression of several lipid metabolism-related genes is increased, and the total triglyceride content is also increased (58). The results of the above experimental studies were consistent with those of the present study. In the yak mammary epithelial cells, overexpression of *TRIB3* decreased triglyceride content, while knockdown of *TRIB3* increased triglyceride content, indicating that *TRIB3* negatively regulates triglyceride synthesis in yak mammary epithelial cells.

## 5 Conclusion

In this study, by interfering and overexpressing the yak *TRIB3* gene, we found that the *TRIB3* gene had a negative regulatory effect on lipid metabolism-related genes *ACACA*, *FASN*, *LPL*, *FABP3*, *ACSL1*, *ACSS2*, *GPAM*, *LPIN1*, *DGAT1*, *PPARg*, *SCAP*, triglyceride content and lipid droplet accumulation. It was also verified that TRIB3 gene could inhibit lipid metabolism in yak mammary epithelial cells by inhibiting Akt signaling pathway. The lipid metabolism characteristics of *TRIB3* in mammary epithelial cells of yaks were preliminically clarified, which laid a theoretical basis for further study of YMECs lipid metabolism, improvement of the regulatory mechanism of milk fat metabolism network and improvement of dairy quality of yaks.

## Data Availability

The original contributions presented in the study are included in the article/supplementary material, further inquiries can be directed to the corresponding author/s.
